# Short Bouts of Gait Data and Body-Worn Inertial Sensors Can Provide Reliable Measures of Spatiotemporal Gait Parameters from Bilateral Gait Data for Persons with Multiple Sclerosis

**DOI:** 10.3390/bios10090128

**Published:** 2020-09-20

**Authors:** Lilian Genaro Motti Ader, Barry R. Greene, Killian McManus, Niall Tubridy, Brian Caulfield

**Affiliations:** 1 CeADAR—Centre for Applied Data Analytics, University College Dublin, Dublin D04 V2N9, Ireland; 2 Kinesis Health Technologies Ltd., Belfield Office Park, Clonskeagh, Dublin D04 V2N9, Ireland; barry.greene@kinesis.ie (B.R.G.); killian.mcmanus@kinesis.ie (K.M.); 3 School of Public Health, Physiotherapy and Sport Sciences, University College Dublin, Dublin D04 V1W8, Ireland; b.caulfield@ucd.ie; 4 Insight Centre for Data Analytics, University College Dublin, Dublin D04 V1W8, Ireland; 5 Department of Neurology, St. Vincent’s University Hospital, Dublin D04 T6F4, Ireland; ntubridy@svhg.ie

**Keywords:** gait analysis, wearable, body-worn sensors, inertial sensors, gait variability, gait symmetry, reliability, walking, multiple sclerosis, short bouts of gait

## Abstract

Wearable devices equipped with inertial sensors enable objective gait assessment for persons with multiple sclerosis (MS), with potential use in ambulatory care or home and community-based assessments. However, gait data collected in non-controlled settings are often fragmented and may not provide enough information for reliable measures. This paper evaluates a novel approach to (1) determine the effects of the length of the walking task on the reliability of calculated measures and (2) identify digital biomarkers for gait assessments from fragmented data. Thirty-seven participants (37) diagnosed with relapsing-remitting MS (EDSS range 0 to 4.5) executed two trials, walking 20 m each, with inertial sensors attached to their right and left shanks. Gait events were identified from the medio-lateral angular velocity, and short bouts of gait data were extracted from each trial, with lengths varying from 3 to 9 gait cycles. Intraclass correlation coefficients (ICCs) evaluate the degree of agreement between the two trials of each participant, according to the number of gait cycles included in the analysis. Results show that short bouts of gait data, including at least six gait cycles of bilateral data, can provide reliable gait measurements for persons with MS, opening new perspectives for gait assessment using fragmented data (e.g., wearable devices, community assessments). Stride time variability and asymmetry, as well as stride velocity variability and asymmetry, should be further explored as digital biomarkers to support the monitoring of symptoms of persons with neurological diseases.

## 1. Introduction

Gait impairment is highly prevalent in multiple sclerosis (MS), as the decline in neural control affects motor functions, and consequently gait, balance and mobility [1,2]. Objective gait measurements enable the assessment of the quality and performance of gait, including gait variability and asymmetry [3], providing important information to complete the neurological evaluation of persons with MS [4,5]. Objective gait assessments are highly sensitive to changes in symptoms, supporting early diagnosis and the evaluation of therapeutic interventions [6,7].

Data collected from wearable devices, equipped with inertial sensors, have been demonstrated to be effective in objective gait assessments, offering a portable and cost-effective solution compared to large or fixed installations [4,5,8]. However, if wearable devices are suitable for ambulatory care, home assessments and community ambulation, the variety of gait assessment protocols can be considered an obstacle for establishing reference values [4]. One challenge is related to the different lengths and durations of mobility tests traditionally used for assessing persons with MS (e.g. Timed 25-Foot Walk, Six Minute Walk test, Timed-Up-and-Go (TUG) test) [9,10,11]. In moving to assessment within non-controlled environments, gait data may be fragmented and non-homogenous [5]. The variations and restrictions on gait assessment protocols (e.g., physical space, time), in both controlled and non-controlled settings, may not provide enough gait data for reliable measures.

One approach to address this challenge is to select short bouts of gait data, representing predefined number of gait cycles, from the full length of a walking task. The analysis of the intrasession reliability can show the effects of length of gait data on the reliability of gait parameters, to identify the spatiotemporal gait parameters that can be used to obtain reliable measures according to the number of gait cycles included in the analysis.

The selected approach focuses on the study of the reliability of spatiotemporal gait parameters from a limited number of gait cycles, which could, therefore, be applied to fragmented data, enabling gait assessments with wearable devices. The main goal of the present study is to define an optimal length of gait data and identify the spatiotemporal gait parameters that can be used to define digital biomarkers for persons with MS.

## 2. Materials and Methods

### 2.1. Participants

Participants were recruited from the neurology outpatient department at St. Vincent’s University hospital, Dublin, Ireland. The inclusion criteria were participants diagnosed with clinically definite relapsing-remitting MS, able to execute two 20 m walking trials safely without a mobility aid. All participants provided informed consent, and ethical approval was obtained from St. Vincent’s hospital research ethics committee.

The present study includes data from 37 participants with MS—mean age 45.1 ± 9.9, height 168.6 ± 9.9 cm, weight 75.5 ± 16.8 kg, 23 females (62%); mean time since diagnosis was 7.4 ± 7.7 years. Each participant received a comprehensive neurological and physical examination including Expanded Disability Status Score (EDSS), which quantifies disability in people with MS ranging from 0 (Normal neurological exam) to 10 (Death due to MS). EDSS steps from 1.0 to 4.5 refer to people with MS who are fully ambulatory (i.e., able to walk 300 m without aid). At the time of the assessment, 13 participants had EDSS score 0, 13 had EDSS scores 1 or 1.5, and 11 had EDSS scores 2 or above. Mean time to complete a TUG test was 7.8 ± 1.7 s, while mean stride velocity was 122.2 ± 15 cm/s and mean stride length 136.6 ± 16.9 cm. A table of clinical information for the participants is available as Supplementary Material.

### 2.2. Procedures

To include measures of gait symmetry, the study protocol was designed to include a collection of bilateral gait. Two inertial sensors were attached to the participant’s right and left shanks, at the midpoint of the anterior shank using dedicated Velcro straps. Sensors sampled at 102.4 Hz and contained a triaxial accelerometer and a triaxial gyroscope. Data were streamed in real time via Bluetooth using dedicated software (Kinesis Gait™, Kinesis Health Technologies Ltd., Dublin, Ireland) and stored for offline analysis.

Participants were instructed to walk at their preferred self-selected pace, starting with their dominant foot (right or left). Each participant completed two trials of the 20 m walking task, in the same day, with a short break between trials.

### 2.3. Data Extraction

Previously published algorithms were selected for the procedures for calibration, data treatment and artefact rejection [12,13,14].

The gait event detection creates a sequence of Initial Contact (IC) and Terminal Contact (TC) points corresponding to the movements of each leg. ICs and TCs represent heel-strikes and toe-offs, respectively, and define the phases of stance (IC to TC) and swing (TC to IC) for each leg.

**Figure 1:**
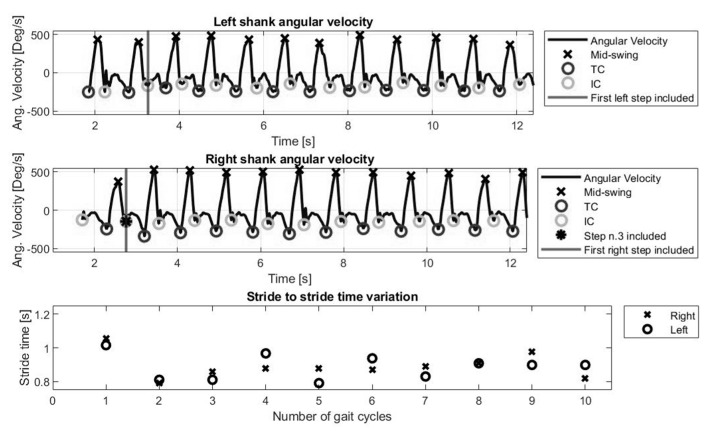
Top panel: medio-lateral angular velocity from left and right shank over time for a 20 m walk task. Bottom panel: stride to stride time variation for one participant (age 49, female, 165 cm height, 60 kg, EDSS 3). Heel-strikes and Toe-offs are referred to as Initial Contact (IC) and Terminal Contact (TC).

The data extraction process ignored the first gait cycle (i.e., first step each leg) and included data from the 3rd IC. Figure 1 represents angular velocity signal and the ICs (i.e., heel strike) and TCs (i.e., toe off) for the full length of a walking task.

Following the gait event detection, ICs and TCs were used to calculate spatiotemporal gait parameters, as described: mean swing time (TC to IC of the same foot, averaged across both legs, in seconds), mean stance time (IC to TC of the same foot, averaged across both legs, in seconds), stride time (IC to IC of the same foot, averaged across both legs, in seconds). Mean step time represents average of times between IC of one foot to IC of the opposite foot, in seconds. Mean single support is the proportion of gait cycle spent on either foot and mean double support is the proportion of gait cycles spent on both feet, averaged across multiple gait cycles. Spatial information was estimated from stride length. Mean stride length (m) and mean stride velocity (cm/s) were calculated and averaged across both legs.

For gait variability, the coefficient of variation (CV) was calculated as the standard deviation (SD) divided by mean values within participants across multiple gait cycles, as a percentage. Gait variability was calculated for stride time, stance time, swing time, step time, single support, and double support.

For gait asymmetry, the Gait Symmetry Index (GSI) represents the difference between right and left divided by the average of right and left values, expressed as a percentage. Minus values indicate left leg asymmetry [15]. Gait symmetry was calculated for stride time, stance time, swing time, step time, stride velocity and stride length. Gait symmetry index is defined by the equation below:

$$GSI=100*\frac{\left(x_{R}-x_{L}\right)}{0.5*\left(x_{R}+x_{L}\right)}$$

where $x_{R}$ and $x_{L}$ are the right and left leg values, respectively, of the given gait parameter.

A table with the definitions of the spatiotemporal gait parameters for the present study is available as Supplementary Material.

### 2.4. Data Analysis

The intrasession reliability was calculated using intraclass correlation coefficients (ICC(2,k)) [16,17]. The ICCs represent the variation in measurements using the same instrument, on the same participant, under the same conditions (test-retest reliability), in the 95% confidence interval [16]. Data analysis was conducted offline using MATLAB (version R2019a, MathWorks, Natick, MA, USA).

Based on the ICC estimate, the reliability of gait parameters was described as “poor” (less than 0.5), “moderate” (between 0.5 and 0.75), “good” (between 0.75 and 0.9) or “excellent” (0.9 and greater) [16].

Participants executed different numbers of steps to complete the walking task. In order to calculate the ICCs for all the spatiotemporal gait parameters, and include all the trials, a minimum number of three and a maximum number of nine complete gait cycles (18 strides, or 9 strides per leg) could be extracted from the full length of the walking tasks. ICCs were then calculated at predefined numbers of gait cycles: 3, 4, 5, 6, 7, 8 and 9.

## 3. Results

The mean time for all the participants to complete each walking task was 16.1 ± 3.2 s. Mean stride velocity (excluding time from recording start to gait initiation) was 146.2 ± 23.5 cm/s, and mean stride length was 135.6 ± 18.5 cm across all trials.

### Intrasession Reliability of Spatiotemporal Gait Parameters

**Figure 2:**
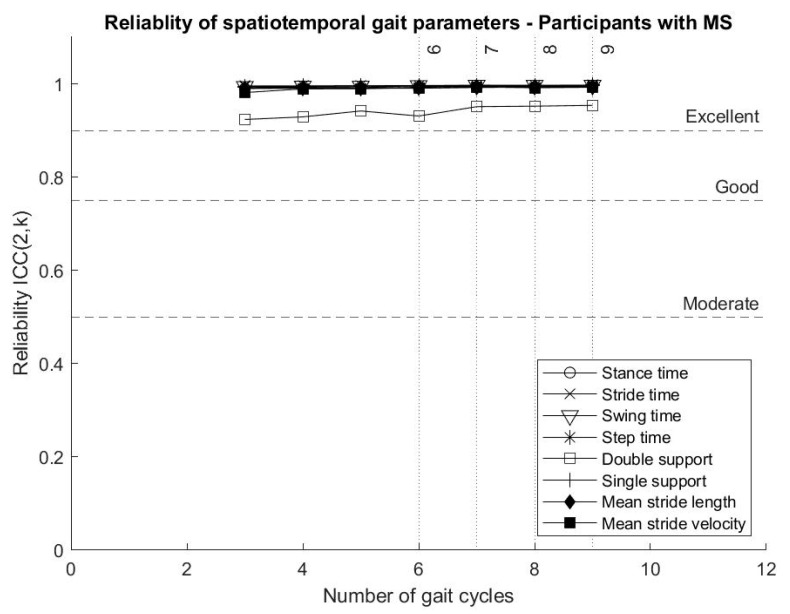
Variation of the reliability (Intraclass correlation coefficients (ICCs)) of spatiotemporal gait parameters for participants with multiple sclerosis (MS) according to the number of gait cycles included in the analysis.

Spatiotemporal gait parameters representing average values across gait cycles reached “excellent” reliability from three gait cycles (six strides, three of each leg), as shown in Figure 2, including mean stance time, mean stride time, mean swing time, mean step time, mean double support, mean single support, mean stride length and mean stride velocity.

**Figure 3:**
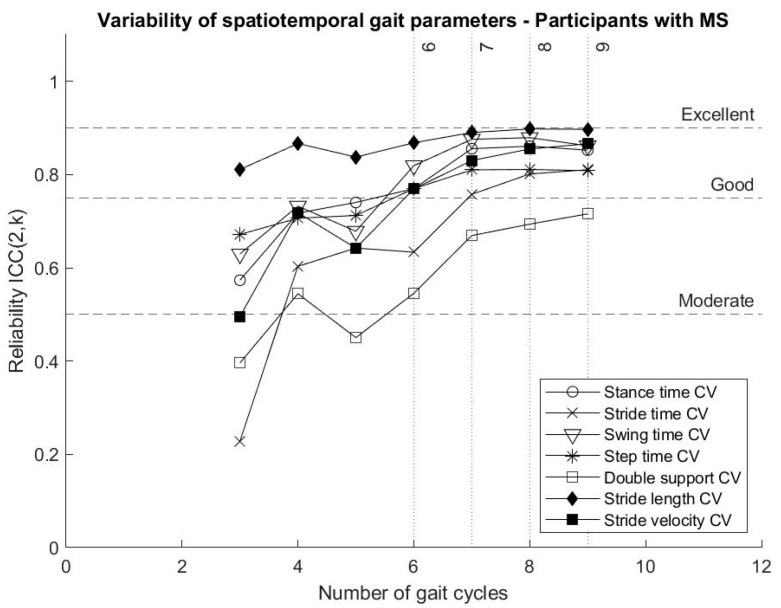
Variation of the reliability (ICCs) of variability of spatiotemporal gait parameters for participants with MS according to the number of gait cycles included in the analysis.

Stride length variability reached “good” variability from three gait cycles, while all the other parameters describing gait variability showed increased reliability when more gait cycles were included in the analysis. Some gait variability parameters, in particular stance time variability, swing time variability, step time variability and stride velocity variability, reached “good” reliability after six gait cycles and tended to continue towards an excellent reliability around nine gait cycles, with an exception of the variability of double support, as shown in Figure 3.

**Figure 4:**
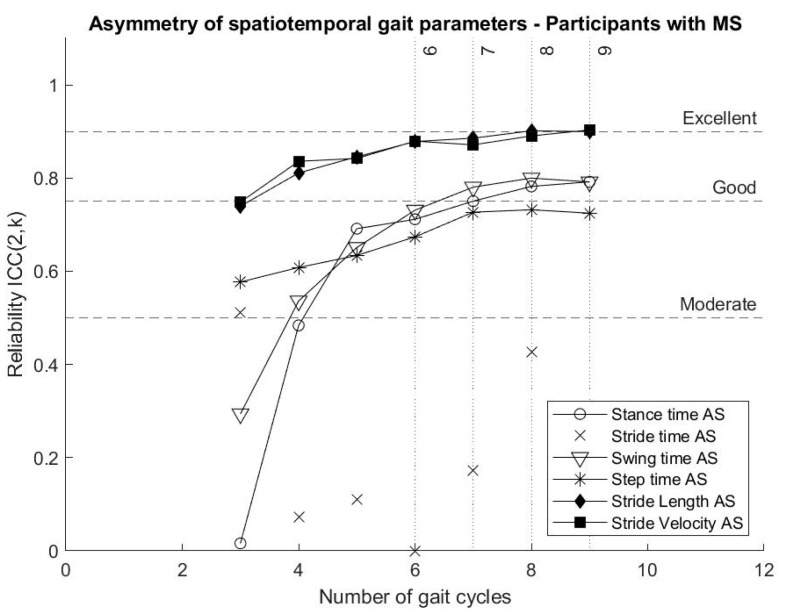
Variation of the reliability (ICCs) of asymmetry of spatiotemporal gait parameters for participants with MS according to the number of gait cycles included in the analysis.

Reliability of parameters describing stride length asymmetry and stride velocity asymmetry reached “good” reliability from four gait cycles, while stance time asymmetry and swing time asymmetry reached “good” reliability from seven gait cycles. Step time asymmetry showed “moderate” reliability even when nine gait cycles were included in the analysis. In the present analysis, the calculation of stride time asymmetry showed “poor” reliability. The reliability of gait asymmetry parameters is presented in Figure 4.

## 4. Discussion

In the present study, we analysed the intrasession reliability of spatiotemporal gait parameters (means, variability and asymmetry) for assessing gait of participants with MS according to a predefined number of gait cycles, representing short bouts of gait data extracted from the full walking task. The main goal of this analysis is to evaluate a novel approach for gait analysis from data collected with wearable devices, supporting gait assessments in ambulatory care and less controlled settings, such as patients’ homes or community ambulation, where gait data are often fragmented [5].

### 4.1. Windowed Approach

The analysis of the reliability of spatiotemporal gait parameters followed the extraction of predefined numbers of consecutive gait cycles from the full walking task, representing short bouts of gait data. This approach has been presented in the literature in order to assess the reliability of measures collected from fragmented data [18]. Another study extracted larger samples of consecutive gait cycles (10 to 60) to compare different conditions of walking tasks (e.g., with turns on the ground and straight walk on a treadmill) as well as the effects of the length of gait data collected [19]. This approach has been applied to identify and recommend an optimal number of strides for reliable measures of gait variability [19], as well as to investigate gait variability over a certain number of gait cycles, and determine the length of gait initiation phase [20]. Results of the present study show that this approach is suitable to enable gait assessments in non-controlled settings using wearable devices, where gait data are often fragmented [5].

As different test conditions can affect the calculated measures [21,22], there is a need for methods enabling the homogenisation of data extraction and calculation in order to obtain reference values [4,5,23]. The analysis of the intrasession reliability for spatiotemporal gait parameters shows that short bouts of gait data could be an effective approach to address this need, supporting gait assessments in ambulatory care. Such an approach could be further explored with the use of wearable devices in controlled and non-controlled environments. However, we argue that for future applications, contextual information should be reported along with the results of gait assessment, such as the length of the walking task and the environment (e.g., indoor or outdoor, home or clinics). We believe this is fundamental to further investigate how the symptoms of neurological diseases evolve over time and how they affect daily routine. This will facilitate the better design of therapeutic interventions matched to disease progression.

### 4.2. Reliability of Spatiotemporal Gait Parameters

In the literature, many authors investigated the reliability of spatiotemporal gait parameters, in particular for measures of gait variability, recommending calculated measures should be carefully reported together with the length of the walking tasks and the test conditions [18,24,25]. Different approaches have been used to estimate reliability, including sampling methods (i.e., bootstrapping) [26], thresholds around the mean value [27], analysis of variance of calculated measures [20] and other specific methods estimating measurement errors through intraclass correlation coefficients [18,19,28,29]. In the present study, the ICCs were calculated to highlight differences according to the number of gait cycles included in the analysis and identify which gait parameters enable reliable measures for gait assessment of persons with MS when a restricted number of gait cycles are available.

The analysis showed that spatiotemporal gait parameters calculated as the average across gait cycles, and representing bilateral gait collected from inertial sensor data, can reach “excellent” reliability from as few as three gait cycles. This result is in line with the literature, describing “good” test–retest reliability for mean stride length, mean stride velocity, mean stance time, mean swing time and mean double support for participants with MS [30].

The present analysis of the reliability of parameters describing gait variability presents stride time variability, stride length variability and stride velocity variability reaching “good” and “excellent” reliability with fewer strides than previous results reported in the literature [24,25]. Including bilateral data, the selected algorithm for data acquisition and gait event detection might have facilitated this result [12,13,31]. This is an important outcome in light of the use of gait variability measures to determine and characterise gait impairment, as well as the potential of sensor-based data to facilitate diagnosis and intervention for persons with MS [7].

### 4.3. Objective Gait Assessment for Persons with MS

One goal of the present study is to determine which spatiotemporal gait parameters can provide reliable gait measurements for short bouts of gait data collected from wearable devices.

As stressed by Frechette et al. (2019), in order to provide relevant clinical data for the assessment of MS related symptoms, it is crucial to focus on gait quality over quantity [5]. Our current study demonstrates that wearable sensors, when collecting bilateral lower limb gait data, can be used to quantify gait from short bouts of data collected, and consequently have the potential to provide reliable measures under free-living conditions to capture the fluctuations of symptoms within a day and over longer periods, as complimentary resources to understand impact and progression of the disease.

A current challenge for wearable technologies is the form factor, enabling them to be worn for long periods of time. Technological limitations such as battery life, weight, shape and material, mean that most wearable devices would only enable data collection for short periods of time, whether used in controlled or in non-controlled settings. One exception is devices strapped to the user’s wrist, which users agree to wear for extended periods of use. However, the wrist worn devices have not been proven to provide reliable measures for gait quality assessment, nor have been studied for patients with mobility impairment. The present study recorded bilateral data from participants’ right and left shanks, which provides complimentary information to evaluate gait asymmetry.

While stride time asymmetry and step time asymmetry have “poor” reliability, results show that gait asymmetry estimated from stance time, swing time, stride length and stride velocity should be further explored, since they provide reliable measures for short bouts of gait data and have the potential to diagnose and monitor symptoms of persons with MS [5].

Our study extends the findings of Strom et al. (2018) on the analysis of the effects of the length of walking bouts on the measures of step-by-step characteristics [32]. The evaluation of the activity of persons with MS and moderate or severe gait impairment for seven days showed that participants were more inclined to walk shorter than longer bouts.

According to Storm et al., 2018, daily living gait is characterised by higher pace compared to studies observed in controlled settings (i.e., laboratories), regardless of the duration of the walking bouts. Concerning the variability of spatiotemporal gait parameters, there was no significant difference between controlled and non-controlled settings, for continuous or intermittent walking. This analysis reinforces our promising result, showing that measures of gait variability and gait asymmetry (in particular of stride length and stride velocity), should be further investigated for their utility in neurological assessment of persons with MS and other neurological conditions.

## 5. Conclusions

The present study shows that short bouts of gait data, representing bilateral gait data collected using inertial sensors, can provide reliable measures for objective gait assessment of persons with MS.

Gait parameters representing average values across gait cycles reached “excellent” intrasession reliability from three gait cycles, while parameters describing gait variability and asymmetry tended to reach higher ICCs when more gait cycles were included in the analysis.

From six gait cycles, stride length variability and asymmetry, as well as stride velocity variability and asymmetry, show “good” reliability and should be further explored with regard to their potential contribution to the early diagnosis and monitoring symptoms of persons with MS. Stride time asymmetry and step time asymmetry do not seem to provide reliable measures and should be reported carefully.

The main contribution of the present study is to demonstrate that short bouts of gait data, including at least six gait cycles of bilateral data, can provide reliable gait measurements for persons with MS, opening new perspectives for wearable devices and digital biomarkers for gait assessment in non-controlled environments, to support the monitoring of symptoms of persons with neurological diseases.

## Supplementary Materials

The clinical profile of the participants included in the present study, and a description of the spatiotemporal gait parameters, are available online at https://www.mdpi.com/2079-6374/10/9/128/s1. The datasets generated and/or analysed during the current study are not publicly available due to data protection regulations in order to protect individual participant’s privacy. Supplementary material includes participants’ clinical profiles and spatiotemporal gait parameters. Additional information can be available from the corresponding author on reasonable request.
