# Supplementary material for: Short Bouts of Gait Data and Body-Worn Inertial Sensors Can Provide Reliable Measures of Spatiotemporal Gait Parameters from Bilateral Gait Data for Persons with Multiple Sclerosis
Source: Biosensors (Basel). 2020 Sep 20;10(9):128. doi: 10.3390/bios10090128 (PMC7558375; doi:10.3390/bios10090128)
Supplement: Supplementary file 1 [file biosensors-10-00128-s001.zip › supplementary-table-participants-profiles.docx]

Table 1 Clinical information of participants

| Participant number | Age | Height (cm) | Weight (Kg) | Gender | EDSS | Duration in years | Average Walk Time (s) for 20m | Average number of Gait Cycles to complete the task | Average Cadence (steps/ min) |
| --- | --- | --- | --- | --- | --- | --- | --- | --- | --- |
| 1 | 46 | 162 | 89.8 | F | 4.5 | 1.333 | 25.47 | 23 | 108.43 |
| 2 | 39 | 169.9 | 81.8 | F | 0 | 2.75 | 13.98 | 14 | 123.03 |
| 3 | 46 | 169.5 | 92.8 | F | 3 | 1.667 | 14.74 | 15 | 119.44 |
| 4 | 41 | 190.5 | 65.7 | M | 1.5 | 0.076 | 14.95 | 13 | 109.67 |
| 5 | 41 | 165 | 64.1 | F | 1 | 2.25 | 15.07 | 15 | 119.38 |
| 6 | 35 | 171.5 | 73.75 | M | 2 | 8 | 18.80 | 18 | 113.87 |
| 7 | 54 | 166.7 | 60.4 | F | 1 | 2.58 | 13.86 | 14 | 123.60 |
| 8 | 59 | 166 | 94.5 | M | 0 | 0.667 | 15.30 | 15 | 121.59 |
| 9 | 47 | 171.5 | 63.6 | M | 1.5 | 16 | 16.85 | 16 | 112.77 |
| 10 | 52 | 175 | 77.8 | F | 1.5 | 8 | 15.57 | 16 | 127.15 |
| 11 | 59 | 164 | 86.4 | F | 2 | 8 | 22.67 | 22 | 119.09 |
| 12 | 45 | 154.2 | 57.4 | F | 0 | 2.917 | 17.11 | 20 | 142.60 |
| 13 | 42 | 181 | 89.05 | M | 0 | 12 | 15.08 | 15 | 122.00 |
| 14 | 49 | 183 | 96.5 | M | 1.5 | 8.583 | 11.81 | 13 | 135.55 |
| 15 | 54 | 161 | 60 | F | 1.5 | 25 | 15.47 | 16 | 124.12 |
| 16 | 38 | 182 | 72 | M | 0 | 8 | 13.15 | 12 | 110.99 |
| 17 | 55 | 173 | 92.65 | M | 2 | 10 | 17.17 | 17 | 118.78 |
| 18 | 48 | 168 | 77.8 | M | 0 | 0.75 | 12.66 | 14 | 132.73 |
| 19 | 57 | 174 | 97.4 | F | 3 | 8 | 17.48 | 17 | 120.20 |
| 20 | 38 | 162.6 | 58.25 | F | 1 | 16 | 16.77 | 16 | 116.87 |
| 21 | 25 | 164 | 67 | F | 2 | 0.135 | 12.22 | 14 | 141.23 |
| 22 | 33 | 158 | 52 | F | 1 | 0.5 | 12.15 | 16 | 159.77 |
| 23 | 28 | 187.96 | 165.7 | M | 0 | 1.667 | 13.40 | 14 | 126.93 |
| 24 | 45 | 163.5 | 68.6 | F | 0 | 9 | 13.63 | 17 | 148.25 |
| 25 | 46 | 163 | 55 | F | 0 | 1.5 | 13.98 | 16 | 140.22 |
| 26 | 42 | 171 | 96 | M | 1 | 6 | 13.77 | 13 | 119.12 |
| 27 | 57 | 160 | 72.4 | F | 1 | 4.083 | 17.29 | 19 | 133.14 |
| 28 | 59 | 154 | 63.5 | F | 0 | 7 | 14.37 | 17 | 140.64 |
| 29 | 37 | 166 | 81 | F | 1 | 3.917 | 13.62 | 16 | 145.44 |
| 30 | 35 | 174 | 100 | F | 1 | 7 | 14.00 | 17 | 145.69 |
| 31 | 49 | 165 | 60 | F | 3 | 19 | 16.93 | 17 | 124.28 |
| 32 | 62 | 160 | 52 | F | 2 | 17 | 15.60 | 17 | 130.78 |
| 33 | 65 | 169 | 64 | F | 2 | 9 | 16.34 | 18 | 130.96 |
| 34 | 37 | 173 | 82 | M | 0 | 1.5 | 12.98 | 14 | 129.49 |
| 35 | 35 | 176 | 83 | M | 0 | 5 | 12.31 | 14 | 138.09 |
| 36 | 58 | 155 | 47 | F | 3.5 | 37 | 24.26 | 23 | 117.88 |
| 37 | 52 | 185 | 90 | M | 0 | 3 | 12.25 | 12 | 124.12 |
