# Supplementary material for: Short Bouts of Gait Data and Body-Worn Inertial Sensors Can Provide Reliable Measures of Spatiotemporal Gait Parameters from Bilateral Gait Data for Persons with Multiple Sclerosis
Source: Biosensors (Basel). 2020 Sep 20;10(9):128. doi: 10.3390/bios10090128 (PMC7558375; doi:10.3390/bios10090128)
Supplement: Supplementary file 1 [file biosensors-10-00128-s001.zip › supplementary-table-spatiotemporal-gait-parameters.docx]

Table 1 Spatiotemporal Gait Parameter Definitions

| **Parameter definition** | **Description** |
| --- | --- |
| **Temporal parameters** |  |
| Recording time (s) | Recording time for entire walking test as recorded using sensors. |
| Walk time (s) | Time participant actually spends in locomotion during test. |
| Number of gait cycles | Number of gait cycles over the course of the walking trial. |
| Number of steps | Number of steps in walking test. |
| Average swing time (s) | Average swing time over all gait cycles, averaged across both legs, swing time is defined as the time between a toe-off point and the heel strike point on the same foot. |
| Average stance time (s) | Average stance time over all gait cycles, stance time is defined as the time between a heel-strike and toe-off point on the same foot. |
| Average stride time (s) | Average time to complete one stride (time between successive heel-strikes), averaged over all gait cycles. |
| Average step time (s) | Average of times between heel-strike of one foot to heel strike of the opposite foot. |
| Average single support | Proportion of a gait cycle spent on either foot. |
| Average double support | Proportion of a gait cycle spent on both feet. |
| **Variability** |  |
| Stride time variability (%) | Coefficient of variation in stride time over the course of walking trial, expressed as a percentage. |
| Stance time variability (%) | Coefficient of variation in stance time over the course of walking trial, expressed as a percentage. |
| Swing time variability (%) | Coefficient of variation in swing time over the course of walking trial, expressed as a percentage. |
| Step time variability (%) | Coefficient of variation in step time over the course of walking trial, expressed as a percentage. |
| Single support variability (%) | Coefficient of variation in the proportion of a gait cycle spent on a single foot over the course of walking trial, expressed as a percentage. |
| Double support variability (%) | Coefficient of variation in proportion of a gait cycle spent on both feet over the course of walking trial, expressed as a percentage. |
| **Symmetry** |  |
| Stride time asymmetry (%) | Gait symmetry index for stride time: difference between right and left divided by average of left and right, expressed as a percentage. Minus values indicate left leg asymmetry. |
| Stance time asymmetry (%) | Gait symmetry index for stance time: difference between right and left divided by average of left and right, expressed as a percentage. Minus values indicate left leg asymmetry. |
| Swing time asymmetry (%) | Gait symmetry index for swing time: difference between right and left divided by average of left and right, expressed as a percentage. Minus values indicate left leg asymmetry. |
| Step time asymmetry (%) | Gait symmetry index for step time: difference between right and left divided by average of left and right, expressed as a percentage. Minus values indicate left leg asymmetry. |
| Stride velocity asymmetry (%) | Gait symmetry index for gait velocity: difference between right and left divided by average of right and left, expressed as a percentage. Minus values indicate left leg asymmetry. |
| Stride length asymmetry (%) | Gait symmetry index for stride length: difference between right and left divided by average of right and left, expressed as a percentage. Minus values indicate left leg asymmetry. |
| **Spatial parameters** |  |
| Average stride velocity (cm/s) | Average walking speed during the walking trial. |
| Stride velocity variability (%) | Coefficient of variation in walking speed over the course of the walking trial, expressed as a percentage. |
| Average stride length (cm) | Mean stride length over the course of the walking trial. |
| Stride length variability (%) | Coefficient of variation in stride length over walking test. |
